# Supplementary material for: Patterns of Intron Gain and Loss in Fungi
Source: PLoS Biol. 2004 Nov 30;2(12):e422. doi: 10.1371/journal.pbio.0020422 (PMC532390; doi:10.1371/journal.pbio.0020422)
Supplement: Table S1 — Also available at http://genes.mit.edu/NielsenEtAl/. (4.3 MB ZIP). [file pbio.0020422.st001.zip › NielsenEtAl/html/652.html]

AN2447.1.NCU08511.1.MG03241.1.FG01481.1


```
 CLUSTAL W (1.82) Multiple Sequence Alignments - Introns Inserted


Sequence 1: NCU08511.1	409 aa
Sequence 2: FG01481.1	403 aa
Sequence 3: MG03241.1	336 aa
Sequence 4: AN2447.1	390 aa
Alignment Length: 410 aa
Number Identitical Residues: 170 aa
Alignment Score (without introns) 9947


MG03241.1 	-------------------~---------------~------------------------
NCU08511.1	MNALVAQYAPATVRISGPP~NSSFLVGYPGISATL0PRIVGKVEIRPSAGFSAPVSVSMV
FG01481.1 	------MAAPASVRVSGPP~NSSFLVGYPGISATL0PRIEGKVEIRPGQGFSMPVPVSLV
AN2447.1  	---------------MAAF0DSAGHCAYS----FQ~PRIEGKVEIRPSVGITAPVNVSLV
          	                ..  .::   . .       .   .. .  .. . : .   :  

MG03241.1 	----------------MAKKHLGTPRRETTDIVGKEHLLFRCPDGKESESVIAMDLPFVL
NCU08511.1	RICLQRRETIHPAAESMAKRHLGTPRRETVDLVGKEVLLFRCVSGKEAESVIAMDLPFQI
FG01481.1 	RICLQRRETIHPDADSIAKRHLGAPRRETTDLVGKEQLLFRCSSGKEAERIIAMDLPFVL
AN2447.1  	TVSLVRRETIHPSADSVAKRRLAPPRKEISDTVGKEMLLFRCPAGRESEEVLSMDLPFVL
          	  .    .:  . :.::**::*..**:*  * **** *****  *:*:* :::***** :

MG03241.1 	FIPFGRGGEETNRRIPPASLQLPSRTAETYYELIVTVQQGASHQNKYHFPLPLQRYDTLS
NCU08511.1	FIPYGRGGEEINRRIPPASLQLPSRIAETYYELVVTVQQGQSTQNRYTFPIPIQRYDTLS
FG01481.1 	FIPYGRGGEESNRRIPPASLQLPSRTAETYYELVVTVQQGHSNQYKYTFPIPLQRYDTLS
AN2447.1  	FIPFGRGGPDASRRVPPASLQLPSRTAETFYEIVVMVQQGHSEQRKYSFPVPIARYDTLS
          	***:**** : .**:********** ***:**::* **** * * :* **:*: ******

MG03241.1 	TFGMYNRPESKVATADNIVTLGISLPRWSYGPLDPITVYIKLAPNPDWLNKAKRVTINKI
NCU08511.1	TFGMYNRPESKVVTADSIVTLGISLPKWSYGPLDPITVFIKLAPNPDMMNKAKKVTINKI
FG01481.1 	TFGMYNKPESKLVTNDNIVHLGINLPRWSYGPSDPITVYIKLSPNLDWLSKAKRVTIDKI
AN2447.1  	TFGMYNRPESAESVSDHLVTLGISLPRWSYGPLDPVSVYVKLSPNPDWISKARKVTINKI
          	******:***   . * :* ***.**:***** **::*::**:** * :.**::***:**

MG03241.1 	TLSIEEEITYNPEGDEPQKKVNRISKHTQPVGTRLPEAGYTTNLGLVFPSRDLRDAEGVM
NCU08511.1	TLAIEEEITFNPEGDEPTKKVNKIAKHTQPVGVKLTETGYVTNLGLVFPARDLRDPDGIV
FG01481.1 	TLTVEEEITYNPEGDEPSKKINKISRQVQVVKTKMPEAGYATNVGLVFPSKDLRDPDGVV
AN2447.1  	TIGIDEEIIYNHEGDEPQRKVKTLTKRTEPIGVKMPPTGFLTNMGLVFPAKDMRDGEGIL
          	*: ::*** :* ***** :*:: ::::.: : .::. :*: **:*****::*:** :*::

MG03241.1 	RRGEPSKFPNHEVTSFTTTSTLYKVEFFLTIK0ASMSSARDITLRQPIVICPMDQQACKE
NCU08511.1	KRGKP-GFPMYEVTSFTTSSTLYKIEFFLSIK0AHMNSARDITLRQPIVICPMDQQACRD
FG01481.1 	KRGKP-QFPLYEVTSFTTTSTLYKIEFYLVIK0VNLSGARDVTIRQPIVICPIDQQACKE
AN2447.1  	PRGKP-GFPTYAVSGFTTTASLYKIEYYLTVK0AHLTSARDIIIRQPIVVCPLDHAGCKE
          	 **:*  ** : *:.***:::***:*::* :* . :..***: :*****:**:*: .*::

MG03241.1 	EIEAIEQAARDASLVDPNHPYTPAKTIVLASHENSLRHLGLCIVGGQKKPLIE
NCU08511.1	EMDAIEQAAKDASKVDPNNPMLPDRVIVRSNERDAIRHLGLCEVGGVKKMLIE
FG01481.1 	EMDAIEQAAKDASHVDPNNPMLPAPTVILANDRDSLRALGLCMVGGQKKPFIE
AN2447.1  	EMEAIEQAAREAAHINPDNPLLPLPSIVRPSDPNGLRHLGVAIVGNQKKPLID
          	*::******::*: ::*::*  *   :: ... :.:* **:. **. ** :*:
```
